# Are body image and eating attitudes, behaviours, and knowledge of parents of pre-schoolers associated with parent feeding practices?

**DOI:** 10.1186/2050-2974-2-S1-O34

**Published:** 2014-11-24

**Authors:** Stephanie R Damiano, Laura Hart, Susan J Paxton, Chelsea Cornell, Fiona Sutherland

**Affiliations:** 1 School of Psychological Science, La Trobe University, Melbourne, Australia

## 

Some parent feeding practices have been linked to disordered eating and higher BMIs in children. Relatively little research has examined factors that influence parent feeding practices. The aim of this study was to identify relationships between the parent feeding practices that have been linked to unhealthy eating patterns in children, and parent body image, eating attitudes, behaviours, and knowledge. Participants were 326 parents of pre-school children from Victoria (97.5% mothers). Parents completed measures of parent body dissatisfaction, body image knowledge, dieting behaviours, and parent feeding practices of their child closest to four years old (58% girls, aged 2-6 years). Knowledge scores were inversely associated with a number of negative feeding practices, including instrumental feeding, and with the frequency of parents reporting negative behavioural intentions to brief vignettes reflecting appearance-based stigma. Parent weight and shape concerns were positively associated with restrictive feeding practices, including restricting high-fat foods in their pre-schooler. Parent dieting was positively related to restricting and controlling feeding practices. Given that such parent feeding practices are associated with long-term risk of weight gain and disordered eating in children, these findings highlight the need for prevention interventions for parents of pre-schoolers.

This abstract was presented in the **Parental Roles in Prevention and Support** stream of the 2014 ANZAED Conference.

